# Supplementary figures and images for: The COVID-19 outbreak in Sichuan, China: Epidemiology and impact of interventions
Source: PLoS Comput Biol. 2020 Dec 28;16(12):e1008467. doi: 10.1371/journal.pcbi.1008467 (PMC7794025; doi:10.1371/journal.pcbi.1008467)

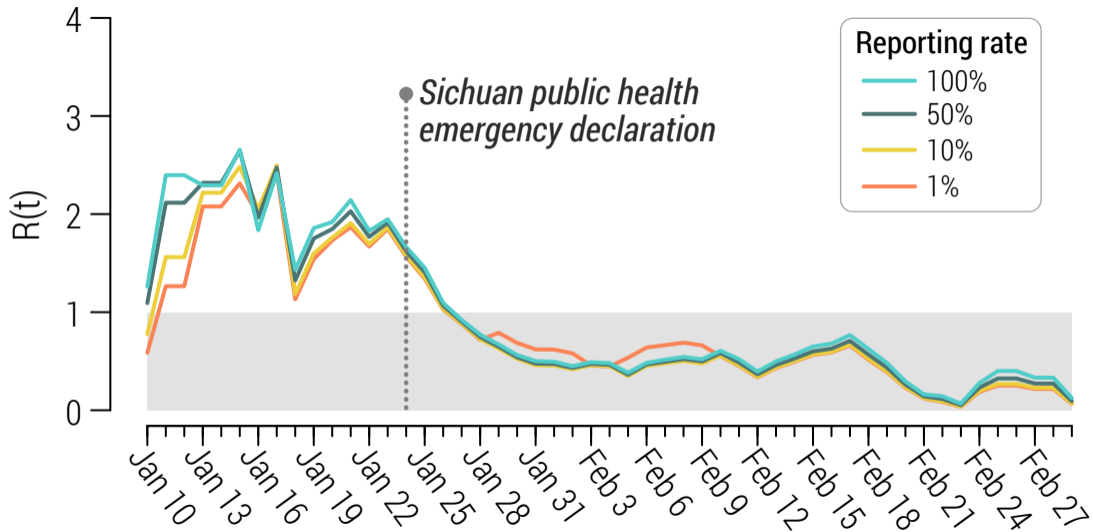

Supplement: S1 Fig — (PDF) [file pcbi.1008467.s001.pdf]

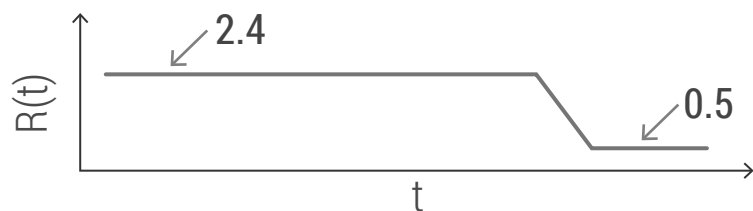

Lockdown starting 4 weeks later  
Reporting rate

100% 50% 10% 1%

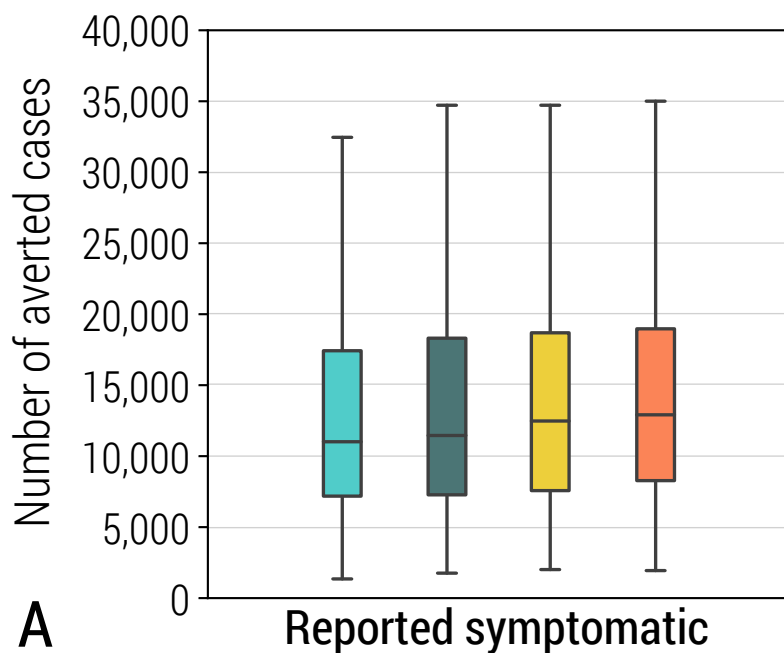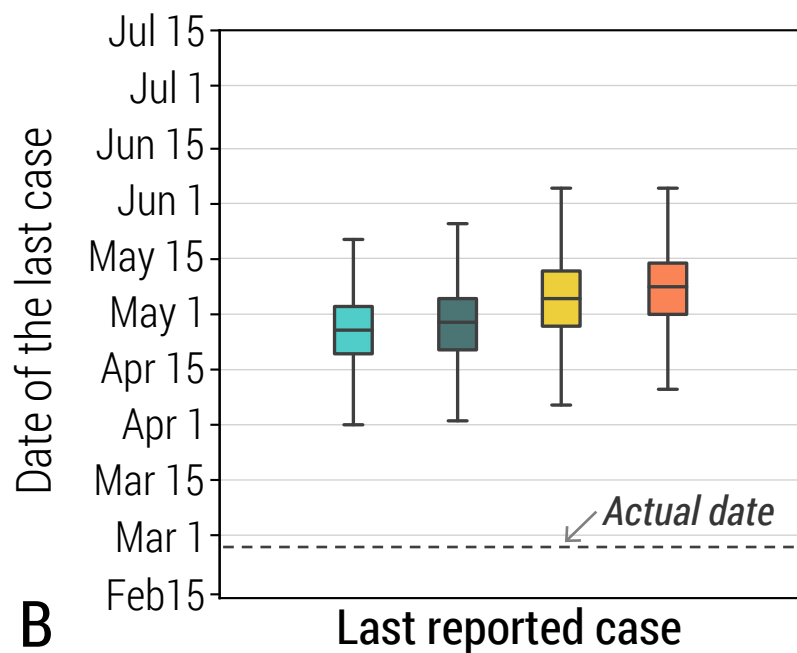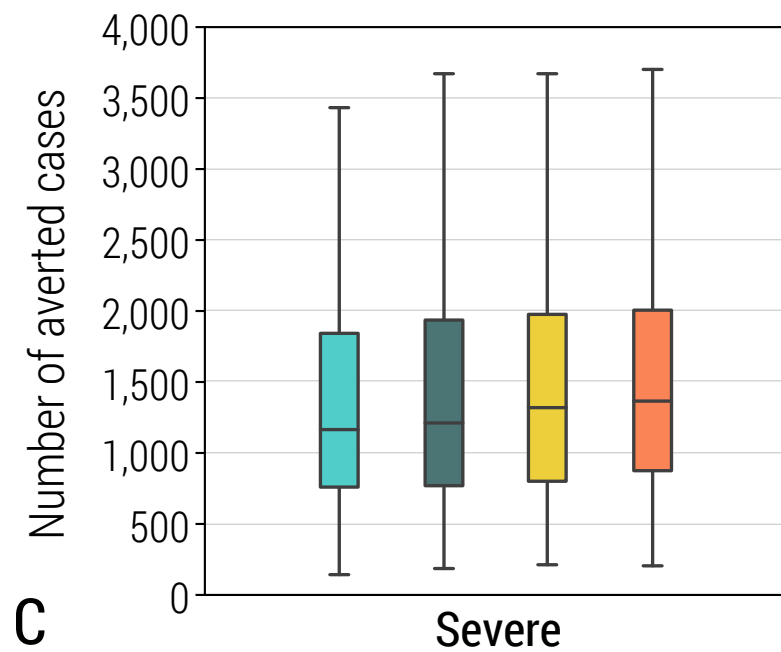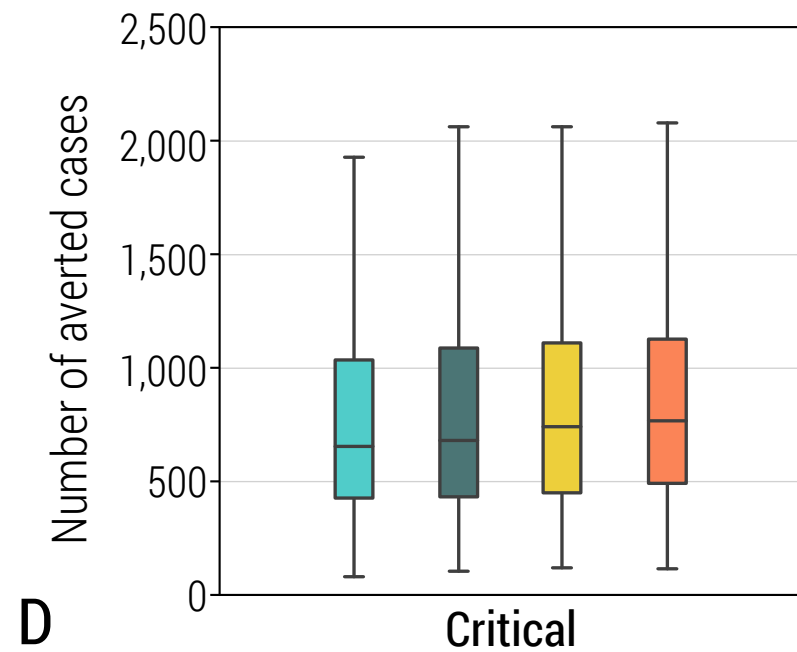

Supplement: S2 Fig — A Estimated number of averted reported symptomatic cases (min, quantile 0.25, median, quantile 0.75, max), should the public health declaration have occurred four weeks later. Estimates are obtained by considering R0 = 2.4 (95% CI: 1.6–3.7) and Rfinal = 0.47 (95% CI: 0.4–0.54); R(t) is assumed to follow a 1-week linear decrease from R0 to Rfinal. Rfinal was estimated over the period from February 1 (i.e., one week after the declaration of the emergency) to the end of the outbreak. Projections are obtained assuming four different values of the reporting rate, namely 1%, 10%, 50%, and 100%. B Same as A, but for the date of the last reported case of the simulated epidemics. C Same as A, but for severe cases. D Same as B, but for critical cases. (PDF) [file pcbi.1008467.s002.pdf]

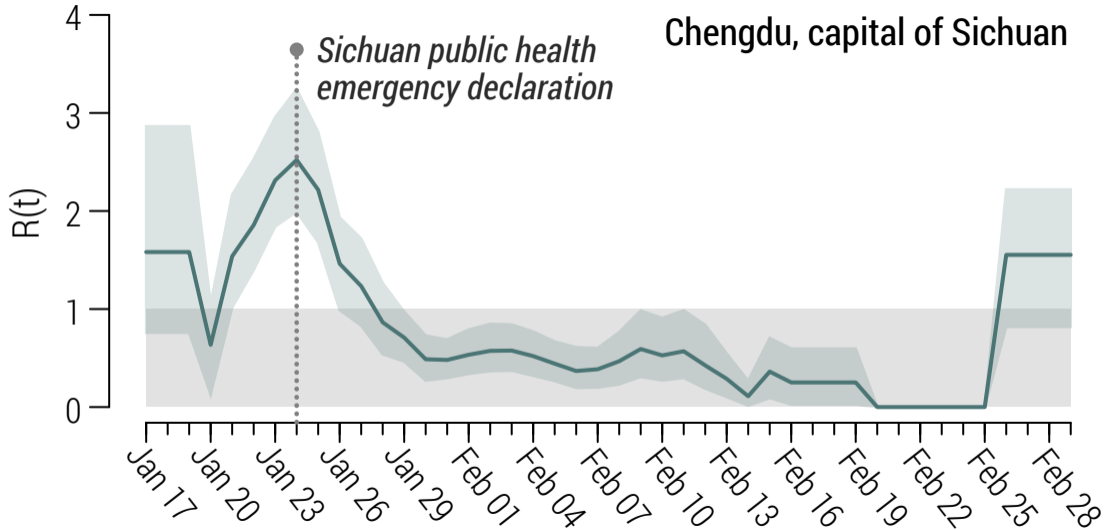

Supplement: S3 Fig — (PDF) [file pcbi.1008467.s003.pdf]

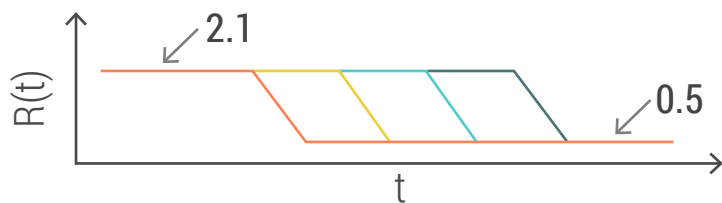

**Lockdown starting:**

one week later

two weeks later

three weeks later

four weeks later

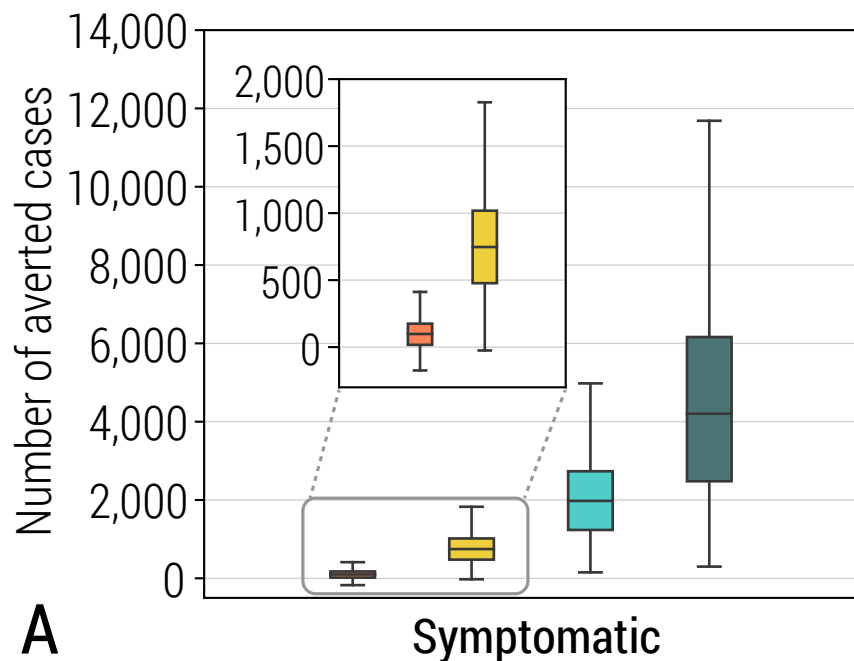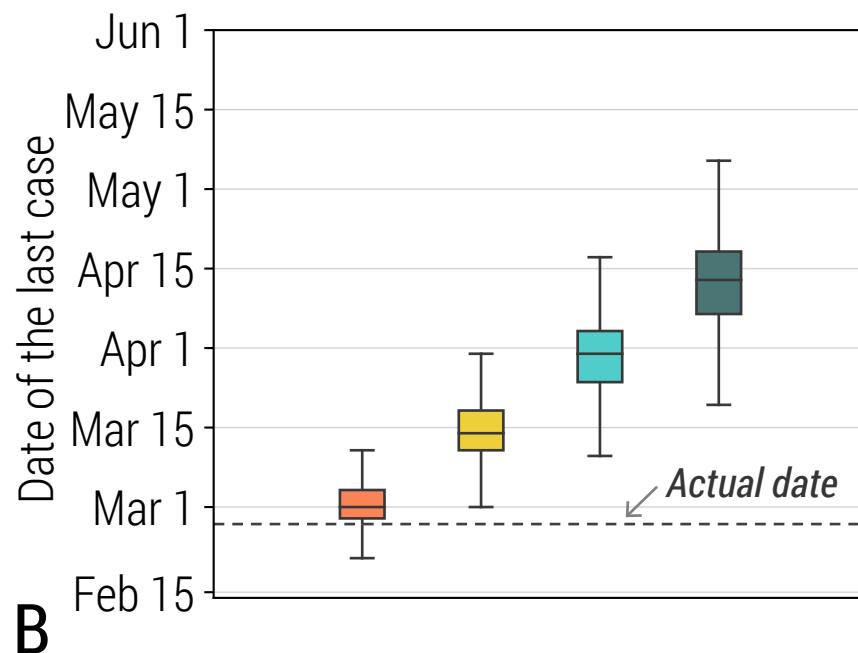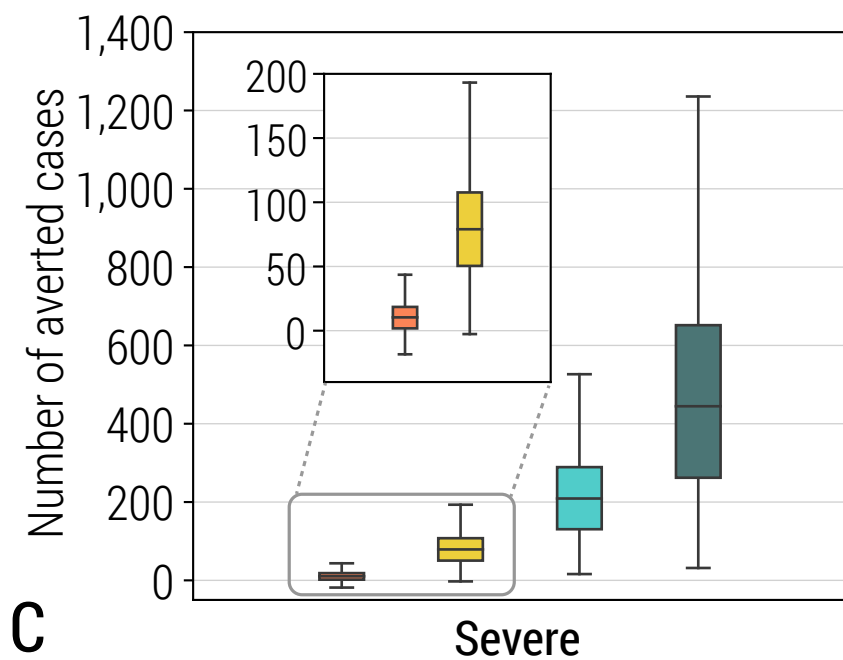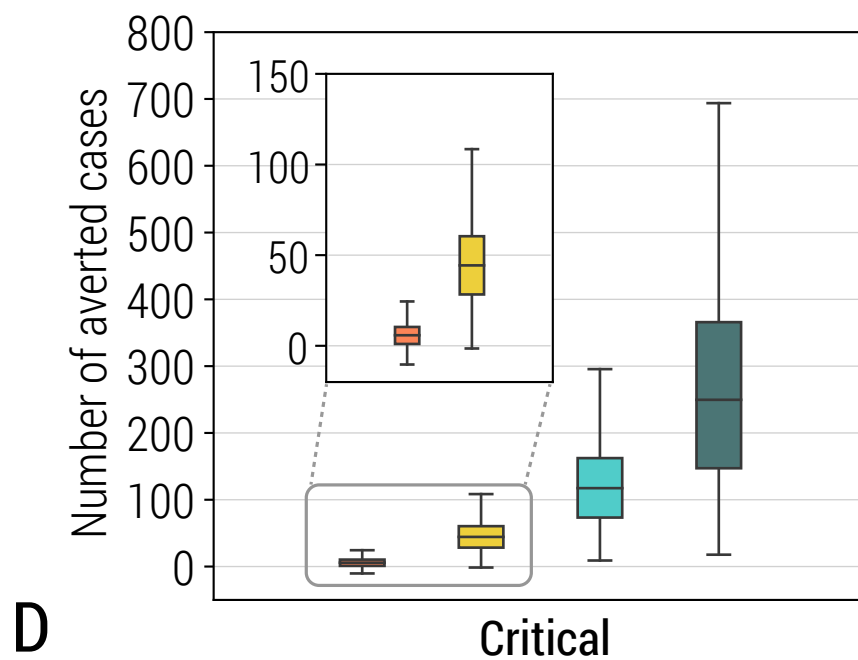

Supplement: S4 Fig — A Estimated number of averted cases (min, quantile 0.25, median, quantile 0.75, max), should the public health declaration have occurred one to four weeks later. Estimates are obtained by considering R0 = 2.1 (95% CI: 1.6–2.7) and Rfinal = 0.47 (95% CI: 0.4–0.54); R(t) is assumed to follow a 1-week linear decrease from R0 to Rfinal. Rfinal was estimated over the period from February 1 (i.e., one week after the declaration of the emergency) to the end of the outbreak. B Same as A, but for the date of the last case of the simulated epidemics. C Same as A, but for severe cases. D Same as B, but for critical cases. (PDF) [file pcbi.1008467.s004.pdf]

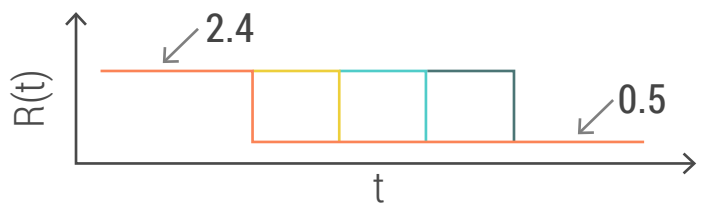

**Lockdown starting:**

one week later

three weeks later

two weeks later

four weeks later

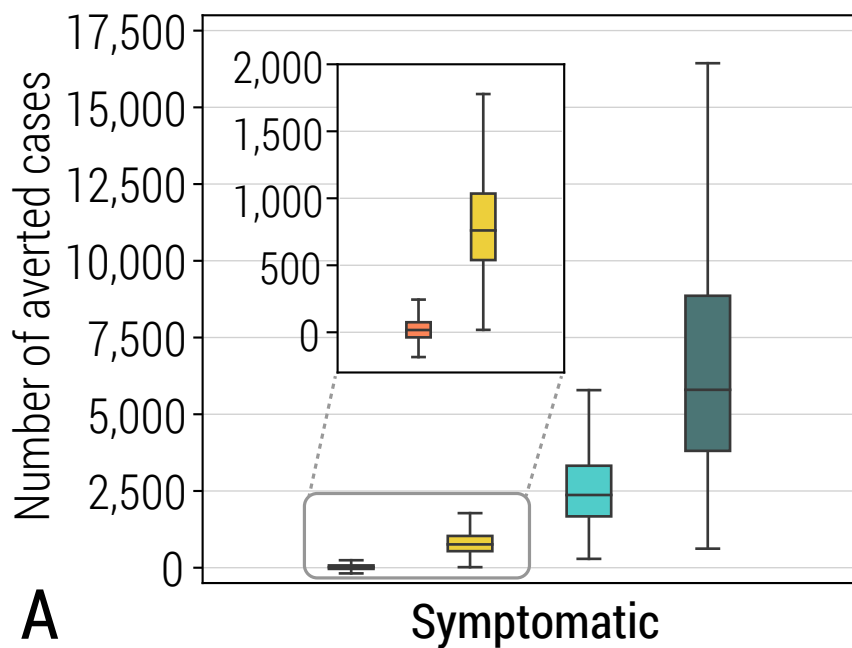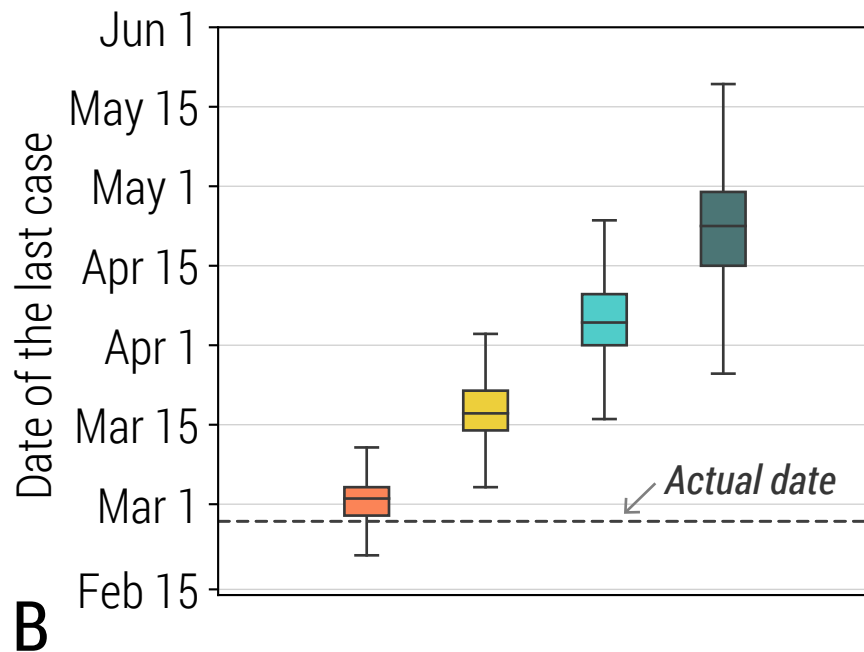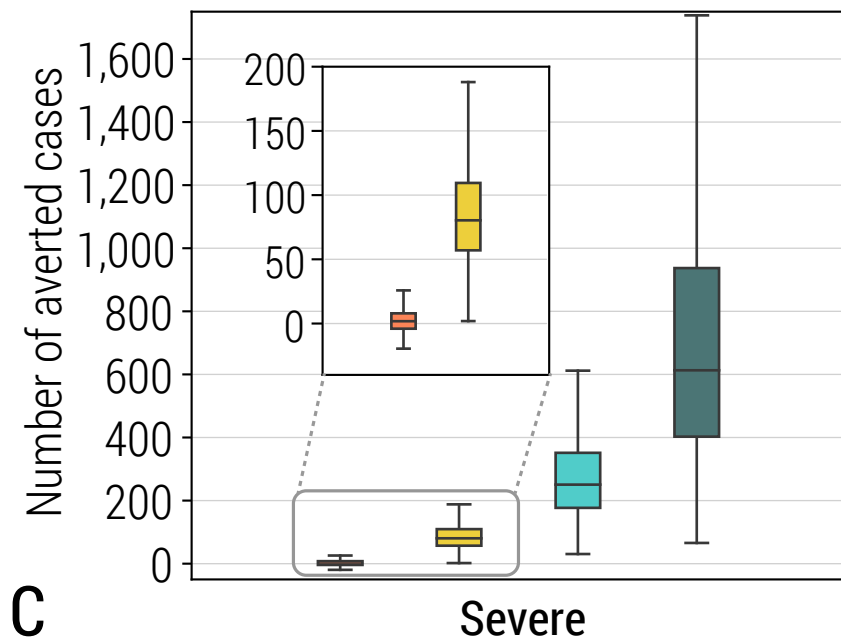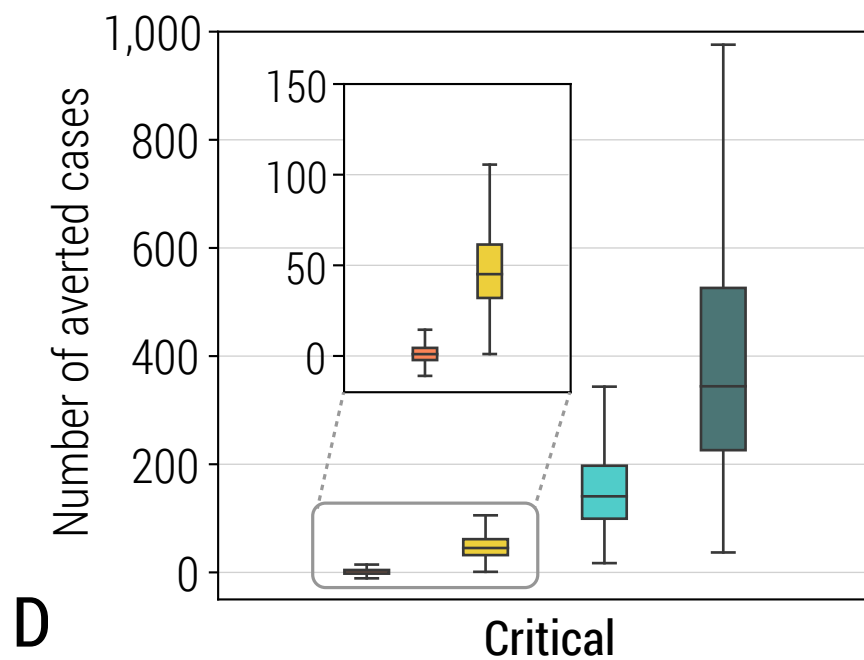

Supplement: S5 Fig — A Estimated number of averted cases (min, quantile 0.25, median, quantile 0.75, max), should the public health declaration have occurred one to four weeks later. Estimates are obtained by considering R0 = 2.4 (95% CI: 1.6–3.7) and Rfinal = 0.53 (95% CI: 0.47–0.60); R(t) is assumed to instantaneously drop from R0 to Rfinal. Rfinal was estimated over the period from January 25 (i.e., the day after the declaration of the emergency) to the end of the outbreak. B Same as A, but for the date of the last case of the simulated epidemics. C Same as A, but for severe cases. D Same as B, but for critical cases. (PDF) [file pcbi.1008467.s005.pdf]
