# Supplementary material for: The COVID-19 outbreak in Sichuan, China: Epidemiology and impact of interventions
Source: PLoS Comput Biol. 2020 Dec 28;16(12):e1008467. doi: 10.1371/journal.pcbi.1008467 (PMC7794025; doi:10.1371/journal.pcbi.1008467)
Supplement: S1 Text — (PDF) [file pcbi.1008467.s007.pdf]

The COVID-19 outbreak in Sichuan, China: epidemiology and impact of interventions

S1 Text

Quan-Hui Liu[1,*], Ana I. Bento[2], Kexin Yang[1], Hang Zhang[1], Xiaohan Yang[3,4], Stefano Merler[5], Alessandro Vespignani[6,7], Jiancheng Lv[1], Hongjie Yu[8], Wei Zhang[9], Tao Zhou[10,*,#], Marco Ajelli[2,6,*,#]

[1] College of Computer Science, Sichuan University, Chengdu, China
[2] Department of Epidemiology and Biostatistics, School of Public Health, Indiana University Bloomington, IN, USA
[3] Department of Engineering and Computer Science, New York University Shanghai, Shanghai, China
[4] Institution of New Economic Development, Chengdu, China
[5] Bruno Kessler Foundation, Trento, Italy
[6] Laboratory for the Modeling of Biological and Socio-technical Systems, Northeastern University, Boston, MA USA
[7] ISI Foundation, Turin, Italy
[8] School of Public Health, Fudan University, Key Laboratory of Public Health Safety, Ministry of Education, Shanghai, China
[9] West China Biomedical Big Data Center, West China Hospital, Sichuan University, Chengdu, China
[10] Big Data Research Center, University of Electronic Science and Technology of China, Chengdu, China

* Corresponding authors: quanhuiliu@scu.edu.cn (Q.-H.L.), zhutou@ustc.edu (T.Z.), and marco.ajelli@gmail.com (M.A.)
# Senior authors

1. Effect of the reporting rate on the estimation of R(t) and of the number of averted cases

To test the effect of the reporting rate on our estimates of the COVID-19 reproduction number in Sichuan, we designed the following experiment. We multiplied the time-series of cases by an arbitrary reporting rate *q*. As no estimate of the reporting rate of COVID-19 in Sichuan is available, used four values of *q*, covering a wide spectrum of values from 1% to 100%. We then applied the methodology presented in the main text for the estimation of R(t) to the four time series (i.e., three time-series assuming reporting rates of 1%, 10%, and 50% in addition to the original time-series considering 100% reporting rate). Except for the first 4 days from January 10 to 13, 2020, when only sporadic cases where reported in Sichuan (5 reported cases in total), the estimated R(t) does not depend on the considered reporting rate (S1 Fig).

A similar experiment was designed to test the robustness of our estimates of the number of averted cases under different scenarios of the timing of the interventions. Here we started from the original time-series of cases *C(t)* and we multiplied it by the same values of the reporting rate *q* described above (namely, 1%, 10%, 50%, and 100%). We then used the renewal equation (as described in the main text) to project the number of cases starting from the time-series *qC(t)* for the four values of *q*. The obtained results are very consistent for different values of the reporting rate (S2 Fig). Indeed, the number of projected infections is different depending on the reporting rate, but the number of reported cases, severe cases, and critical cases does not. In fact, unless the depletion of susceptible individuals becomes relevant (which is not the case here, considering that we project less than 40,000 cases in a population of about 86,000,000 individuals), the renewal equation does not depend on the reporting rate, but rather on the value of R(t).

2. Spatial distribution of cases in Sichuan

The number of COVID-19 confirmed cases for each of the 21 prefectures of Sichuan is reported in S1 Table. The only prefecture reporting more than 100 cases is Chengdu (the capital and most populous city of Sichuan) with 144 reported cases. For Chengdu, the number of cases is enough to allow us to estimate R(t) using the methodology presented in the main text and applied to the entire Sichuan Province. The estimated R(t) has a comparable qualitative and quantitative trend with that estimated for the entire Sichuan (compare S3 Fig with Fig 2). The only remarkable differences are: i) in Chengdu, the reproduction number during the first week was lower than in Sichuan; and ii) in the last 4 days of February, we observe a fluctuation of R(t) above the epidemic threshold linked to a small cluster of cases.

3. Sensitivity analyses on the projected number of cases

We report here the results of two sensitivity analyses showing alternative counterfactual scenarios. The first sensitivity analysis considers R_0, as estimated over a 2-week time period before the declaration of the public health emergency (instead of the last one, as assumed in the main text). Therefore, mean value of R_0 used to project the number of cases is 2.1 instead 2.4 as in the baseline analysis. The obtained results are shown in S4 Fig and commented in the main text.

The second sensitivity analysis considers an instantaneous drop from R_0 to R_{final} at the time of the declaration of the public health emergency (instead of to a linear decrease as for the baseline analysis presented in the main text). The obtained results are shown in S5 Fig and commented in the main text.
